# Depression and Self-Concept: Personality Traits or Coping Styles in Reaction to School Retention of Hispanic Adolescents

**DOI:** 10.1155/2011/151469

**Published:** 2011-04-28

**Authors:** Rebecca A. Robles-Piña

**Affiliations:** Department of Educational Leadership and Counseling, Sam Houston State University, P.O. Box 2119, Huntsville, TX 77341, USA

## Abstract

The purpose of this study was to investigate whether depression and self-concept could be construed as personality characteristics and/or coping styles in reaction to school retention or being held back a grade. The participants in this study were 156 urban Hispanic adolescents, ages 12–18, and of these, 51 or 33% had been retained in school. Students who had been retained reported a lower self-concept score, higher GPA, and higher rates of depression, and they were more likely to be male than students who had not been retained. The findings of this study indicated that self-concept was a personality characteristic that, due to its malleability, is also a coping style in regards to retention with this Hispanic adolescent population.

## 1. Introduction

Stressful events and circumstances such as early school retention, depression, and low self-concept can have far-reaching consequences in children's lives. Finding positive ways of helping children cope with these stressors is critical for their education. Integral to finding ways is determining whether depression and self-concept are personality characteristics, because if these two psychological constructs are part of personality, they are more likely to be part of a students' typical way of responding and less likely to be responsive to interventions. On the other hand, if depression and self-concept are found to be linked to students' means of coping with retention, then they are more likely to be affected by contextual factors and thus be much more likely to be responsive to school interventions. 

Of the variables considered in this study (retention, self-concept, depression, and grade point average—GPA), the literature indicates that some of them can be stressful, others are used as coping, and some of them interact with one another. For example, it is well documented that school retention, being held back a grade in school, is a stressful event that negatively affects academic progress [1–4] and enhances the possibility of behavioral problems in children [5–7]. Depression was considered as a variable in this study because of the high depression rates found for Latino adolescents who have higher rates than for other ethnic groups [8, 9]. 

Self-concept and self-esteem were also considered variables, and often times, they are used are used interchangeably in research, and this is also the case in this research. Both earlier studies [10–12] as well as most recent studies have indicated that low self-esteem predicts depression [13, 14]. Whereas self-concept is a factor that has promise in acting as a means of coping against the effects of depression, unfortunately, the positive and negative impacts of self-concept on retention are inconclusive [1, 3, 5]. 

A gap in the literature exists in determining whether the aforementioned variables (depression, self-concept, and GPA) are related to school retention. Moreover, there is a lack of literature on whether these variables act as personality traits which are more enduring or whether they work as a means of coping against school retention. Further, the literature is nonexistent in exploring this research with Hispanic adolescent populations. Thus, the purpose of this study was to investigate whether any of the psychological variables (depression and self-concept) discussed acted as personality traits or a means of coping in reaction to retention in urban Hispanic adolescents.

## 2. School Retention

Despite the empirical research on the deleterious effects of retention, there are national school policies within the last decade that have called for its implementation [15–17]. Ironically, these policies have been implemented in efforts to improve student learning and to improve the quality of education for all children, but due to the retention policies that coexist, certain ethnic groups and many children are left behind in their education. The social and educational variables that are impacted by retention are numerous. Jimerson et al. [5] have indicated that when students are retained because they lack social maturity and behavior skills, in the subsequent year, the problems have been exacerbated. Other social indicators that are affected by retention are drug and alcohol use and teenage pregnancy [18, 19]. Future behavior is also impacted as students who have been retained are more likely to be unemployed, be on welfare, or be in prison as adults [20]. 

Contrary to popular assumptions, retention has not prevented academic failure. In fact, it has been found that students who have been retained are twice as likely as non-retained peers to repeat a grade the second time around [21]. Retention also increases the likelihood that students will drop out of school by 78% [22]. An indication of how retention affects graduation is evidenced by the fact that only 50% of ethnic minority students graduate with their peers [23]. A most recent study that investigated the predictors for retention of Hispanic students in the first grade indicated that the best predictors were being young for the grade and parents' low sense of responsibility for their children's adjustment to school [24]. In other words, Hispanic students were retained because they did not have preschool experience and entered school without the literacy skills needed, and parents did not question teacher's decisions about the retention of their child. 

 Earlier research had indicated that perhaps early-grade retention was better than later retention, but most recent research has not supported the effectiveness of that notion [25]. In fact, these same authors indicated that early grade retention has been “one of the most powerful predictors of later school withdrawal” (Brantley et al., [26, page 452]). 

The emotional stress of being retained can be quite devastating to students. For example, Byrnes and Yamamoto [3] and Sevener [4] found that young children perceived retention as punishment and expressed feelings of fear, anger, and sadness. Another example of emotional stress due to retention was found when Anderson et al. [2] surveyed sixth grade students. They found that students rated retention as the third most stressful event, with going blind and the death of their parents rated as the first and second stressors. Interestingly, the same authors conducted the survey 20 years later and found that retention was rated as the most stressful event with going blind and the death of their parents second and third.

## 3. Depression, Personality, Stress, and Coping

Depression, the most common form of emotional problems experienced during adolescence, can be characterized by feelings of sadness, anxiety, fear, guilt, anger, contempt, and confused thinking [27]. Whether depression can be identified as a personality trait has been the subject of much research, partly due to a lack of agreement and difficulty in finding a concise meaning for personality [28]. 

Even the father of personality, Gordon Allport, classified over 50 meanings of personality [29]. Many years later, Maddi [30] defined personality as a “stable set of characteristics and tendencies that determine those commonalities and differences in the psychological behavior (thoughts, feelings, and actions) of people and that have continuity in time” (page 10). Contemporary studies on personality indicate that there are three fundamental terms used to define personality: traits, states, and types [31]. Personality traits can be interpreted as the enduring characteristics that distinguish one person over another and some words that are often used to describe someone's personality are “outgoing,” “passive,” “extrovert,” and others. States, on the other hand refers to how a person responds to an event that is temporary. And types refer to personality traits that are clustered into different types or categories, such as the *Myers-Briggs Type Indicator* (MBTI, [32]). 

Not only there is no agreement about the definition of personality and whether depression is a personality trait, state, or type, there is also debate on whether depression is a mood disorder. Some earlier researchers have indicated that personality and mood affect have significant parallels [33]. Contemporary researchers have presented a variety of models to explain the relationship between personality and depression and suggest that personality can be a precursor, integral part, mediator, or a consequence of a mood disorder [34]. Specifically, the models indicate that (a) mood disorders and personality occur at the same time, (b) personality is a precursor to mood disorders, (c) personality increases the risk of the mood disorder, (d) personality influences the mood disorder, and (e) the mood disorder has an enduring impact on personality. 

Regardless of whether depression is treated as a trait, state, or type, it often appears on assessments of personality for measuring depression in psychopathology in adults, such as the *Minnesota Multiphasic Personality Inventory-2* [35]. Further, depression is used as a subscale in assessing clinical and nonclinical populations of children with the *Behavior Assessment for Children-2 *[36]. Moreover, depression is used in the measurement of personality in nonclinical adult populations with the *NEO Personality Inventory *[37] and is specifically measured using the neuroticism scale. Reviews of the *NEO Personality Inventory* [38, 39] including the neuroticism scale indicate strong evidence of reliability and validity. Hence, based on the aforementioned discussion of personality and for the purposes of this study, depression will be defined as a personality characteristic. 

Depression has been determined to affect particular ethnic groups more than other groups. Earlier studies have indicated that Hispanic students report more depression than other ethnic groups [40]. Information from a nation-wide report indicates that this trend continues with Hispanic students (34%) reporting more depression than Whites (26.5%), and African Americans (28.8%) [41].Within-group comparisons of Hispanic adolescents in rural and urban areas have also been high with 33% rates reported for rural areas [42, 43] and 36% for urban areas [44]. A most recent study investigating the effect of culture and context on depression in Mexican American youth indicates that Latino youth continue to be at a higher risk for depression compared to youth from other ethnic groups [8]. 

Failure to address depression in adolescents can lead to an increase in suicides as the most important predictor for suicide is depression. According to the Centers for Disease Control [41], 19% of high school students have seriously considered suicide and another 8.8% have made one or more attempts. A gender comparison indicated that females (23%) more than males (8.8%) have considered a suicide attempt and also make more attempts. An ethnic comparison indicated that the suicide rate is higher for Native Americans, White teens are more likely to consider a suicide attempt; however, most Hispanic teens are more likely to make an attempt. In 2001, Olvera reported that adolescents of Mexican American descent were at a higher risk for suicidality than other ethnic groups. Important to remember is the fact that pathways to the development of depression and other social and emotional variables may be different for diverse ethnic groups [45]. Ultimately, it is especially important to understand that shameful experiences, such as low grades, retention, and/or rejection by a romantic partner can also trigger depression and ultimately suicide [46, 47].

## 4. Self-Concept, Personality, Stress, and Coping

Self-concept has sometimes been referred to as a personality characteristic. Recent work by personality researchers has indicated that personality traits such as the big five (neuroticism, extraversion, conscientiousness, agreeableness, and openness) are “core” characteristics that are not subject to change, while self-concept is defined as a malleable personality characteristic [48]. They define it as such because self-concept can be influenced by context, life events, and environmental factors. Further, Marsh et al. have indicated that self-concept is a multidimensional construct and have determined that the causal effects of personality on behavior are more likely to be mediated to some extent by self-concept. 

Several studies have concluded that low self-concept is associated with depression in an inverse manner; in other words, as self-concept decreases, depression rates go up [10–12, 44]. Modrcin-Talbott and others found in their study of 77 adolescents a correlation of *r* = −.46 between depression and self-concept, and Robles-Piña et al. found a similar correlation of −.47. 

Siegel et al. [11] studied other variables such as gender, racial/ethnic group, and physical appearance in investigating the relationship between depression and self-esteem. The researchers found that overall Hispanic adolescents were more depressed and had lower self-esteem than other racial/ethnic groups. Additionally, Siegel et al. [11] and Robles-Piña et al. [14] found that Hispanic females had more negative feelings about their bodies than White and African American female adolescents and that these feelings contributed to higher levels of depressive symptoms and lower self-esteem. Depressed mood, self-esteem, and body image have had some common variance between both recent immigrants and more acculturated Hispanic girls. Siegel et al. have suggested that Hispanic females' negative feelings about their bodies might have led to consequences of marginalization. Specifically, Hispanic females became more marginalized because they lacked the personal self-esteem of Black girls and the academic opportunities of White girls. A most recent study by Robles-Piña et al. [44] indicated that the following predictors: (a) self-concept, (b) past feelings of depression, (c) retention, and (d) being female were most likely to predict depression in Hispanic urban adolescents.

## 5. Self-Concept and Retention

Few studies have been conducted to investigate the positive and negative effects that retention have on students' self-concept [49]. In their nationwide Swiss study, Bonvin et al. found no evidence to support that retention had negative social or emotional effects. The extant studies about the effect that self-concept has on retention are divided into three different schools of thought. There are studies that have indicated that grade retention may have a negative effect on self-concept and leads to social and emotional problems, especially in the long term [1, 3, 5, 50]. Conversely, there are also a few researchers who have found positive effects of grade retention on the self-concept of students who have been retained [51–53]. Even so, there is also some research that has indicated no difference between students' self-concept on those retained and those who have been promoted, especially in the short term [5, 52, 54, 55]. In summary, the literature on the effects that retention has on the self-concept of children is mixed and merits further investigation.

## 6. Method

### 6.1. Participants

The participants in this study were 156 Hispanic adolescents, ages 12 through 18, including (63%) males and (37%) females who were attending schools in a large urban setting in the Southeastern region of Texas. The data were collected from a purposeful sample of 5 different school districts in a large metropolitan area, where 20 middle schools were identified and 5 were randomly selected for participation. Once a consent form was received, the student's assent was also requested. The overall rate of response was 78%. The age range was from 13 to 18 years of age with the majority of students in the 16-years-of-age category. The students were in grades sixth through twelfth with the majority in eighth grade (28%). The majority of students reported grades of Bs (40%) and Cs (34%). Further, the majority of students (105, 67%) had not been retained, with (51, 33%) indicating that they had been retained. Of those that had been retained, the majority had been retained in Kindergarten and first grade (43, 28%) with the next peak in retention rates in the eighth and ninth grades (8, 5%).

### 6.2. Measures

There were three instruments used in this study to measure variables of interest. *The Center for Epidemiological Studies Depression Scale* (CES-D) [56] was used to measure levels of depression. The *Piers-Harris Children's Self-Concept Scale* (PHCSCS) [57] was used to measure self-concept. Finally, a general information questionnaire was used to collect educational, demographic, and psychosocial data.

The *CES-D* [56] is a self-report inventory developed to measure the presence of depressive symptoms, with particular emphasis on measuring the affective component of depression. The *CES-D* was originally developed by the National Institute of Mental Health to measure depressive symptomatology in community samples; however, it is now also used in medical settings [26]. An internal consistency coefficient for the scores of this sample was .92. The *CES-D* includes 20 items, written at a third-grade reading level, that measure depressed mood, feelings of guilt, worthlessness, helplessness and hopelessness, loss of appetite, sleep disturbance, and psychomotor retardation. Respondents are instructed to indicate the degree to which they have experienced these symptoms during the preceding week. Some sample items are “My sleep was restless” and “I felt sad.” Responses are made on a 4-point scale ranging from 0 (none of the time) to 3 (most or all of the time). The total scores can range from 0 to 60. Total scores can be classified into four levels: 0–15.5 = no depression, 16–20.5 = mild depression, 21–30.5 = moderate depression, and 31–60 = severe depression. To our knowledge, the use of the categories mentioned, has not been used in previous research. 

The *Piers-Harris Children's Self-Concept Scale* (PHCSCS) [57] is a standardized, 80-item, self-report measure of children's feelings about themselves. The measure consists of six factors: I = behavior, II = intellectual and school status, III = physical appearance and attributes, IV = anxiety, V = popularity, and VI = happiness and satisfaction. The PHCSCS can be used with children in grades 4 through 12 or ages 8 through 18. It may be group or individually administered. Sample item questions are “I am a leader in games and sports” and “I am dumb about most things.” A Cronbach's alpha coefficient of .85 for total self-concept was reported for this sample. 

The information questionnaire used in this study requested the following self-reported information related to demographics, educational, and psychosocial dimensions: (a) age, (b) gender, (c) grade placement, (d) grades, (e) retention history, and (f) earlier feelings of sadness in the early grades. Trainers were prepared for the collection of data. Before collection of data in schools, three persons had achieved an interrater reliability coefficient of .92 in administrating, scoring, and interpreting instruments. Finally, the research project was approved by the university's Internal Review Board.

## 7. Results

Before conducting an MANOVA, assumptions testing determined that the observations in participants were independent of each other; the data were not all normally distributed; however, both ANOVA and MANOVA are robust to moderate violations of normality [58] as long as it is not due to outliers; there was homogeneity of covariance by examining Box's test, and the relationship among all pairs of dependent variables were linear. Thus, it was determined that an MANOVA using a Pillai's trace due to unequal samples sizes [59] could be conducted to make comparisons between retained and non-retained students on psychosocial emotional indicators (depression, GPA, and self-concept). It was determined that there were statistically significant differences on several variables (*ν*(3, 141), 3.141 = .856, *P* = .01). Students who had been retained reported (a) a lower self-concept score (*M * = 49.07, SD = 12.60) than those who had not been retained (*M * = 56.66, SD = 12.91), (b) higher GPA (*M * = 2.75, SD = .84) than students who had not been retained (*M * = 2.27, SD = .79), and (c) higher rates of depression (*M * = 21.05, SD = 10.15) than those who had not been retained (*M * = 14.68, SD = 10.48) (see Table 1). Using a Chi-square for analysis, males (38) reported having been retained more often than females (14) (see Table 1). 

A discriminant stepwise analysis was conducted to determine whether four variables (self-concept, depression, GPA, and gender) would predict whether a child would be retained or not retained in school. One function was determined statistically significant with a Wilk's Lambda, Λ = .89, *χ* ^2^(4, *N* = 142) = 21.15, *P* < .001, explaining 14% of the function variability in school retention. Two variables were entered into the function: self-concept and grade-point ratio, respectively. The variables excluded from the analysis were depression scores and gender, because they did not add to the prediction.  Table 2 presents the standardized function coefficients and correlation coefficients. Evaluation of the standardized discriminant function coefficients reveals that self-concept had the highest loading with  .86 followed by grade-point average with a −.70. The function was labeled grades and feelings of self. Classification results revealed that the original grouped cases were classified with 69% accuracy (see Table 3). Accuracy by each group was 65% accuracy for school retention and 35% for no retention. The cross-validated results supported original accuracy levels with 68% correctly classified overall. Group means for the function indicated that those who were retained had a function mean of −.59, and those who were not retained had a mean of .27. These results indicated that students who have been retained in school had the lowest self-concept and the lowest grades.

Analysis of the differences between retained and non-retained students on the self-concept sub-scales indicated several findings (see Table 4). Students who had been retained indicated more behavioral problems, lower intellectual and school status, anxiety, and popularity than students who had not been retained.

## 8. Discussion

The purpose of this study was to determine if depression, self-concept, and GPA would predict being held back a grade for Hispanic urban adolescents. A further purpose was to determine if self-concept and depression acted as personality factors or as a means of coping in reaction to retention. A limitation of this study was the inability to design a study where the findings could be determined to be causal; this study can only determine associations. The sampling procedure and statistical analyses only permitted for relationships to be established. A future study with longitudinal data is recommended. Further, retention rates and grades were collected from students by self-report, and this may have affected the veracity of the data provided. 

Initial findings indicated that out of the 156 Hispanic adolescents, 51 or 33% had been retained in school with a majority retained in kindergarten and first grade. Students who had been retained reported a lower self-concept, higher GPA, and higher rates of depression, and they were more likely to be male. Due to a low number (*n* = 8) of students who had been retained in later grades, there was an inability to find differences between these students and those who had been retained in early grades.

The finding that indicated that males were more likely to be retained in school is consistent with the literature that indicates that male graduation rates are lower than female graduation rates [60]. Reports of lower self-concept and higher rates of depression for retained students is inconsistent with a most recent research study in Switzerland that indicates that there are no social and emotional effects to children who have been retained [49]. The findings of this study are more consistent with the literature that indicates the stressful nature of retention as indicated by low self-concept [2–5]. 

**Figure 1:**
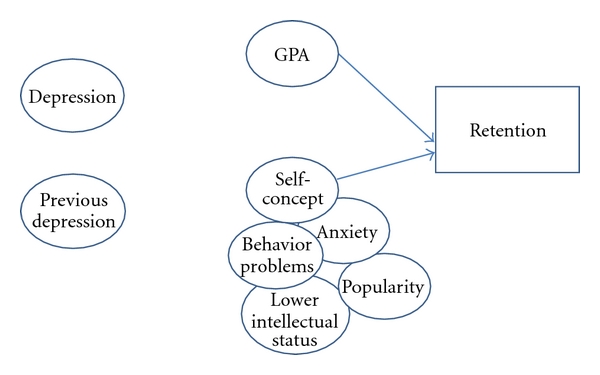
Self-concept as personality and coping style in reaction to retention.

Figure 1 is used to graphically describe the relationships that will be discussed below. Depression, previous depression, GPA, and self-concept were all entered as predictor variables to predict and determine the criterion—retention. Using the literature as a basis, depression was tested as a personality factor [35, 37–39] and self-concept as a personality factor that is more flexible and responsive to environmental factors [48]. We found that depression, a personality characteristic, did not statistically and significantly affect retention. Our findings did indicate that self-concept, a more malleable personality factor that is influenced by external factors, was the greatest predictor of students' retention status. GPA was also entered and explained a very small portion of the variance on whether a student is retained or not. In an earlier study, the findings indicated that self-concept was the most important predictor in predicting depression [44] in Hispanic adolescents, and in this present study, self-concept continues to be the most important predictor in predicting retention. Thus, it appears that self-concept is an enduring, yet malleable, personality factor that not only predicts depression but also retention in Hispanic adolescents. 

In order to determine if self-concept was used as a means of coping, the subscales of self-concept were compared for retained and non-retained students. The following aspects of self-concept were found to contribute to the likelihood of being retained: (a) behavioral problems, (b) lower intellectual and school status, (c) more anxiety, and (d) more popularity than non-retained students. An analysis of the items that contributed to the endorsement of the subscales mentioned led to further insights. For example, in the behavior subscales, students endorsed items related to hating school, getting into a lot of fights, experiencing family disappointment, getting the family in trouble, and doing bad things. On the subscale, lower intellectual and school status, students failed to endorse such items as being smart, being an important person, having good ideas, finishing school work, volunteering in school, and endorsed being dumb about most things. On the anxiety subscale, students endorsed items such as being nervous, giving up easily, worrying about tests, feeling left out, and being unhappy. Interestingly, students who had been retained felt more popular than non-retained students and endorsed items such as I have many friends and I am popular with the girls. These findings support the notion that self-concept is a coping style and that addressing factors noted in the subscales can help prevent or mitigate the effects of retention.

 Implications of these findings on interventions are several. First, educators need to assess the self-concept of students who have been retained. Second, attention to school interventions that address the problems noted in the subscales is important [61]. Third, it is important to find out why retained students who reported higher grades than non-retained students subsequently reported low academic and intellectual status. Further, it is important to find out why students who feel they are popular are also students who have been retained. Fourth, school policies regarding retention need to be revisited. As identified by Roeser and Eccles [62], school policies can lead to emotional stress in students. School retention policies need to be revisited from the perspective of mental health outcomes and well-being perspectives, rather than solely from academic outcomes. In summary, we can assist students to cope with the stress of retention by integrating our understanding of permanent aspects of personality as well as those nonenduring factors such as self-concept.

## Figures and Tables

**Table 1:** Means and standard deviation of predictor variables as a function of school retention and no school retention.

Predictor variables	School retention M/SD	No school retention M/SD
Pier Harris self-concept	48.57/13.19	58.11/12.46
Grade point average	2.75/.83	2.26/.79
CESD depression scale	2.13/1.15	1.60/.94
Gender	Males	Females

**Table 2:** Correlation of predictor variables with discriminant function (function structure matrix) and standardized discriminant function coefficients.

Predictor variable	Correlation with discriminant functions	Standardized discriminant function coefficients
Piers Harris Self-concept	0.86	0.74
Grade point average	−0.70	−0.53

**Table 3:** Classification analysis for school retention and no school retention.

Actual group membership		Predicted group membership	
School retention *n* (%)	No school retention *n* (%)	School retention *n* (%)	No school retention *n* (%)
46 (32%)	98 (68%)	30 (65%)	16 (35%)
		29 (29%)	70 (71%)

Note. 70% of original group correctly classified.

68.3% of cross-validated grouped cases correctly classified.

**Table 4:** Comparisons of students who where retained with non-retained students on the piers harris self-concept sub-scales.

Subscales	Retained (49)		Non-retained (97)	
	Mean	SD	Mean	SD
Behavior = 1*	8.27	2.73	5.70	2.51
Intellectual and school status = 2*	9.18	2.96	10.37	2.69
Physical appearance and attributes = 3	8.04	2.97	8.76	2.52
Anxiety = 4*	6.97	4.05	4.31	2.86
Popularity = 5*	6.27	2.64	4.90	1.72
Happiness and satisfaction = 6	6.35	2.10	6.37	1.41

Note. Statistically significant <.00.
